# A systematic review of randomised controlled trials examining the therapeutic effects of adult bone marrow-derived stem cells for non-ischaemic dilated cardiomyopathy

**DOI:** 10.1186/s13287-016-0441-x

**Published:** 2016-12-09

**Authors:** Yi Lu, Yiqin Wang, Menglu Lin, Jiale Zhou, Zi Wang, Meng Jiang, Ben He

**Affiliations:** Department of Cardiology, Renji Hospital, School of Medicine, Shanghai Jiaotong University, 160 Pujian Road, Shanghai, 200127 China

**Keywords:** Bone marrow-derived stem cell therapy, Non-ischaemic dilated cardiomyopathy, Cardiac systolic function, Mortality rate, Follow-up

## Abstract

**Background:**

Certain early-phase clinical trials have suggested that bone marrow-derived stem cell transplantation might improve left ventricular function in patients with non-ischaemic dilated cardiomyopathy (NIDCM), whereas others trials have revealed no benefit from this approach. We sought to evaluate the therapeutic effects of bone marrow-derived stem cell therapy on NIDCM.

**Methods:**

We searched the PubMed, Embase, and Cochrane Central Register of Controlled Trials (CENTRAL) databases (through February 2016) for randomised controlled clinical trials that reported on bone marrow-derived stem cell transplantation for patients with NIDCM with a follow-up period ≥12 months. The co-primary endpoints were changes in mortality rate and left ventricular ejection fraction (LVEF); the secondary endpoints were changes in the 6-minute-walk test (6MWT) and left ventricular chamber size. Seven trials involving bone marrow-derived stem cell therapy that included 482 patients satisfied the inclusion and exclusion criteria.

**Results:**

Subjects who received bone marrow-derived stem cell therapy exhibited a significant reduction in mortality rate (19.7% in the cell group vs. 27.1% in the control group; 95% confidence interval (CI) –0.16 to –0.00, *I* ^2^ = 52%, *p* = 0.04). Bone marrow-derived stem cell therapy tended to produce LVEF improvement within 6 months (1.83% increase; 95% CI –0.27 to 3.94, *I* ^2^ = 74%, *p* = 0.09) and significantly improved LVEF after mid-term (6–12 months) follow-up (3.53% increase; 95% CI 0.76 to 6.29, *I* ^2^ = 88%, *p* = 0.01). However, this therapy produced no significant benefit in the 6MWT (*p* = 0.18). Finally, the transplantation of increased numbers of stem cells resulted in no observable additional benefit with respect to LVEF.

**Conclusions:**

Bone marrow-derived stem cell therapy might have improved prognoses and appeared to provide moderate benefits in cardiac systolic function at mid-term follow-up. However, this therapy produced no observed improvement in exercise tolerance.

## Background

Non-ischaemic dilated cardiomyopathy (NIDCM) is a major cause of heart failure (HF) and the primary indication for heart transplantation [1, 2]. The prevalence of this disease tends to increase with population age, and survival has improved due to advances in pharmacological treatment and implantable devices [3]. However, current therapeutic approaches are palliative in that they cannot directly address the underlying problem of the loss of cardiac tissue [4].

Although multiple studies have recently investigated the potential role of stem cell therapy for NIDCM [5–11], in the burgeoning field of cell therapy for HF, NIDCM has been studied much less frequently than ischaemic cardiomyopathy (ICM). Two small trials found that the administration of bone marrow mononuclear cells (BMMNCs) via an intracoronary route produced a small but significant increase in left ventricular ejection fraction (LVEF) [7, 12]. Recently, a study demonstrated that an intracoronary injection of CD34+ cells was associated with mid-term improvement in ventricular function, exercise tolerance, and long-term survival in a randomised trial of patients with NIDCM [5]. However, two other recent publications [8, 9] reached contradictory results regarding the impact of stem cell therapy on NIDCM patients.

The questions of whether intracoronary cell therapy improves left ventricular morphology and function and how persistent this effect may be remain unresolved. Our goal was to systematically summarize randomised controlled trials (RCTs) by conducting a meta-analysis of the impact of bone marrow-derived stem cell therapy on NIDCM patients that can serve as a reference for further clinical trials and management.

## Methods

### Review question and study protocol

This review assessed RCTs that examined the effects of adult bone marrow-derived stem cell transplantation on NIDCM.

### Eligibility criteria and search strategy

We searched the PubMed, Embase, Science Citation Index, and the Cochrane Central Register of Controlled Trials (CENTRAL) databases for papers published through February 2016. Additionally, we searched the American College of Cardiology, American Heart Association, European Society of Cardiology, and internet-based sources of information regarding clinical trials in cardiology for RCTs from 2000 to 2016, without language restrictions. The following keywords were used: “non-ischemic cardiomyopathy”, “dilated cardiomyopathy”, “stem cell”, “bone marrow cell”, “mesenchymal stem cell”, “haematopoietic stem cells”, and “progenitor stem cell”. The initially selected citations were screened at the title/abstract level. Potentially relevant publications were retrieved, and the complete manuscripts were assessed for compliance with these inclusion criteria: 1) a prospective comparison of stem cell therapy versus control for NIDCM patients; 2) an intention-to-treat analysis; 3) reported data that include changes in LVEF before and after intervention; and 4) a follow-up period >12 months. The exclusion criteria were: 1) irretrievable or unclear data; 2) lack of a control group or a control arm that also received stem cells; 3) duplicated reports; and 4) on-going or unpublished studies.

### Data extraction

Study features were extracted, including study design, outcome definitions, imaging modalities, patients’ baseline characteristics, and procedural data. Changes in the mortality rate, LVEF, left ventricular end-systolic volume (LVESV), left ventricular end-diastolic dimension (LVEDD), and walk test from baseline to follow-up were collected. If remodelling volume was reported based on multiple imaging techniques, the volume with the smaller standard error was chosen for analysis [13]. The mortality rate, which was evaluated at the longest available follow-up time, was also collected. Two authors independently performed the literature search and data extraction. In cases with missing or unclear data for the primary or secondary endpoints, the primary authors were contacted on at least two different occasions separated by an interval of at least 3 weeks.

### Outcomes

The co-primary endpoints were changes in mortality rate and LVEF from baseline to follow-up. The secondary efficacy endpoints were changes in the 6-minute-walk test (6MWT) and left ventricular chamber size. Data were separated into short-term (<6 months) and mid-term (6–12 months) subgroups.

### Quality assessment

Quality assessment was performed by utilizing the Cochrane Collaboration’s tool for assessing risk of bias criteria in accordance with the PRISMA statement [14].

### Statistical analyses

Statistical analyses were performed using RevMan 5.3. For continuous parameters, weighted mean differences (WMDs) were calculated using mean values, their corresponding standard deviations, and treatment arm sizes. Moreover, data were analysed using 95% confidence intervals (CIs). The percentage of variability across studies attributable to heterogeneity beyond chance was estimated using *I* ^2^. *I* ^2^ > 75% indicated high heterogeneity. A fixed-effect model was adopted when heterogeneity existed (*I* ^2^ > 75%); otherwise, a random-effect model was used. A forest plot was used to display the WMD and 95% CI for each study. A two-sided *p* value <0.05 was regarded as significant.

### Sensitivity analyses and publication bias

Given the small number of studies in our pooled analysis, we tested the robustness of our results with sensitivity analyses that omitted one study at a time. Potential publication bias was assessed using the Egger test and is presented graphically via Begg funnel plots [15, 16], which were adjusted using rank correlation tests.

## Results

### Search results

**Fig. 1:**
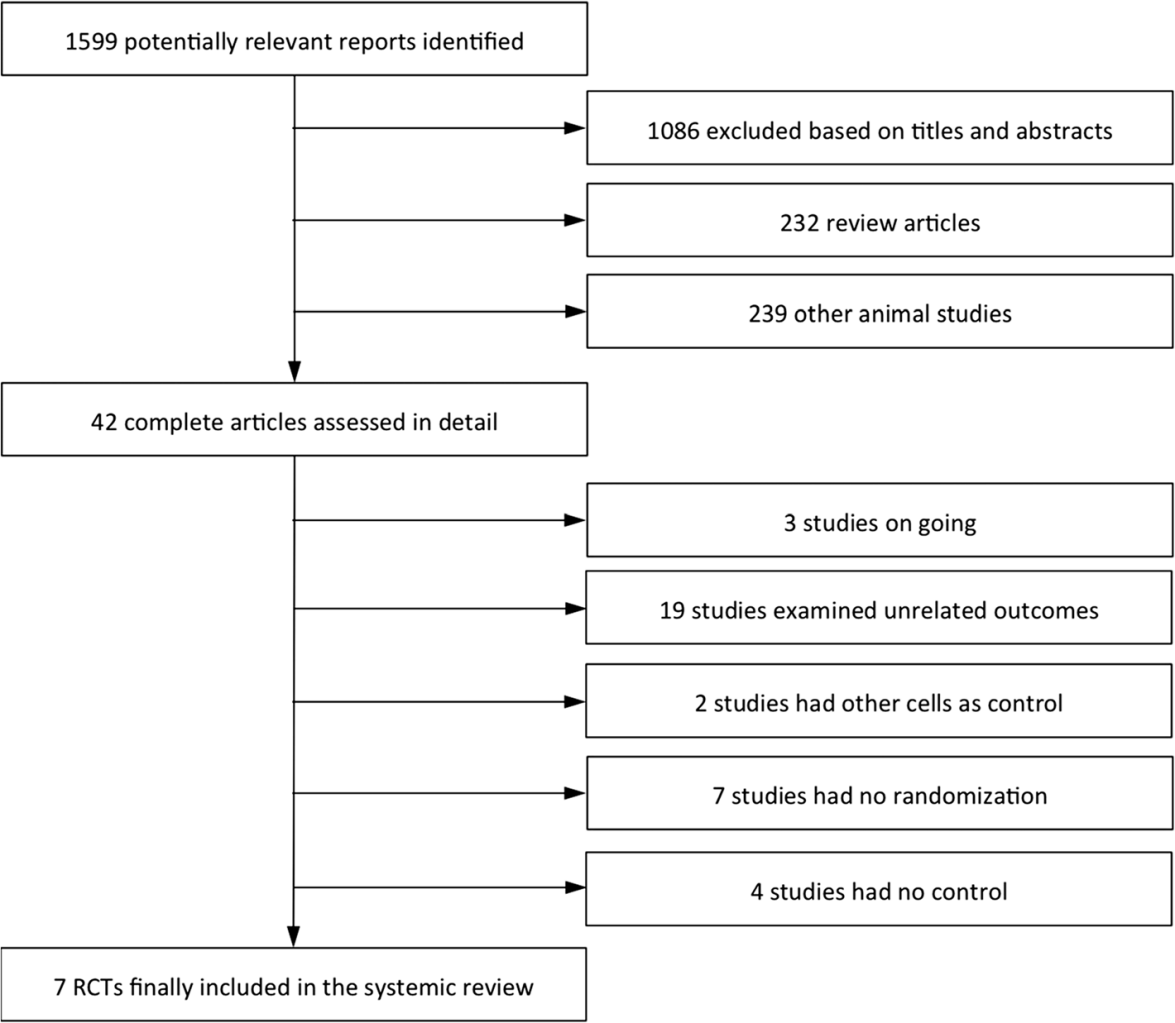
Review process. This scheme diagrams the reviewing process. *RCT* randomised control trial

Figure 1 summarizes the review process. Of the 1599 articles retrieved during the initial search, 1086 articles were excluded at the title and abstract level because they were not relevant, 232 articles were reviews, and 239 articles were animal studies. Among the articles retrieved in complete form, three articles described on-going studies, 19 articles were excluded due to unrelated outcomes, two articles utilized other cells as the control, seven articles described studies with no randomization, and four articles described studies with no control group or a control arm that also received stem cells. Thus, seven trials with a total of 482 patients and a follow-up period ≥12 months were eligible for inclusion [5–11].

### Study characteristics

Table 1 describes the methodological quality of the included RCTs. Table 2 summarizes the main features of the included studies. The included trials were published between 2010 and 2015. The pooled analysis included 227 control patients and 254 patients who received stem cell therapy. The sample sizes for stem cell therapy ranged from 23 to 160, with the longest follow-up ranging from 12 to 60 months. Of the seven identified trials, five trials were multicentre studies [5, 6, 9–11]. Table 3 presents patient and procedural characteristics for the included RCTs. Within each study the control and treatment groups were similar with respect to mean age and gender (*p* > 0.05). The mean patient age in these trials ranged from 45 to 60 years. The injection routes were intracoronary for all trials except for that performed by Henry et al. [10], who used an intramyocardial route. BMMNCs or CD34+ cells were the major cell types involved in these trials. Considerable heterogeneity in the number of cells used for cell therapy was observed (range = 0.35 × 10^8^ to 2.95 × 10^8^ cells).

**Table 1:** Quality assessment

Source	Random sequence generation	Allocation concealment	Blinding of participants and personnel	Blinding of outcome assessment	Incomplete outcome data
Bocchi EA et al. 2010 [11]	L	L	H	H	L
Vrtovec B et al. 2011 [6]	L	H	H	L	L
Vrtovec B et al. 2013 [5]	L	H	H	L	L
Martino H et al. 2015 [9]	L	L	L	L	L
Henry TD et al. 2014 [10]	L	L	L	L	L
Sant’Anna RT et al. 2014 [8]	L	L	H	L	L
Seth S et al. 2010 [7]	L	H	H	H	L

*L* low risk, *H* high risk (Cochrane Handbook for Systematic Reviews of Interventions, Version 5.1.0)

**Table 2:** Main feature of included studies

Source	Sample size, *n*	Follow-up duration, months	Setting	Study design	Primary endpoint
Bocchi EA et al. 2010 [11]	23	22	Multicentre	RCT	LVEF
Vrtovec B et al. 2011 [6]	45	12	Multicentre	RCT	LVEF, exercise capacity
Vrtovec B et al. 2013 [5]	110	60	Multicentre	RCT	LVEF, LVEDD
Martino H et al. 2015 [9]	160	12	Multicentre	RCT	LVEF
Henry TD et al. 2014 [10]	29	12	Multicentre	RCT	Safety
Sant’Anna RT et al. 2014 [8]	30	12	Single centre	RCT	LVEF
Seth S et al. 2010 [7]	85	36	Single centre	RCT	LVEF, mortality

*LVEDD* left ventricular end-diastolic dimension, *LVEF* left ventricular ejection fraction, *RCT* randomised control trial

**Table 3:** Patients and procedural characteristics

Source	Mean age, years	Cell type	Cell transplanted, *n*	Route of injection	Baseline LVEF, %
Bocchi EA et al. 2010 [11]	51 ± 15	BM-CD34+	0.96 × 10^8^	IC	21.8 ± 3.8
Vrtovec B et al. 2011 [6]	52 ± 8	BM-CD34+	1.23 ± 0.23 × 10^8^	IC	25.9 ± 4.6
Vrtovec B et al. 2013 [5]	53 ± 8	BM-CD34+	1.13 ± 0.26 × 10^8^	IC	24.0 ± 4.0
Martino H et al. 2015 [9]	51 ± 11	BM-MNCs	2.36 (1.76–2.95) × 10^8^	IC	24.0 ± 9.6
Henry TD et al. 2014 [10]	60 ± 16	BM-MNCs	1.65 (0.35–2.95) × 10^8^	IM	25.8 ± 7.0
Sant’Anna RT et al. 2014 [8]	48 ± 9	BM-MNCs	1.06 × 10^8^	IC	25.1 ± 4.0
Seth S et al. 2010 [7]	45 ± 15	BM-MNCs	1.68 ± 0.96 × 10^8^	IC	22.5 ± 8.3

*BM* bone marrow, *IC* intracoronary, *IM* intramyocardial, *LVEF* left ventricular ejection fraction, *MNC* mononuclear cell

### Co-primary outcomes

**Fig. 2:**
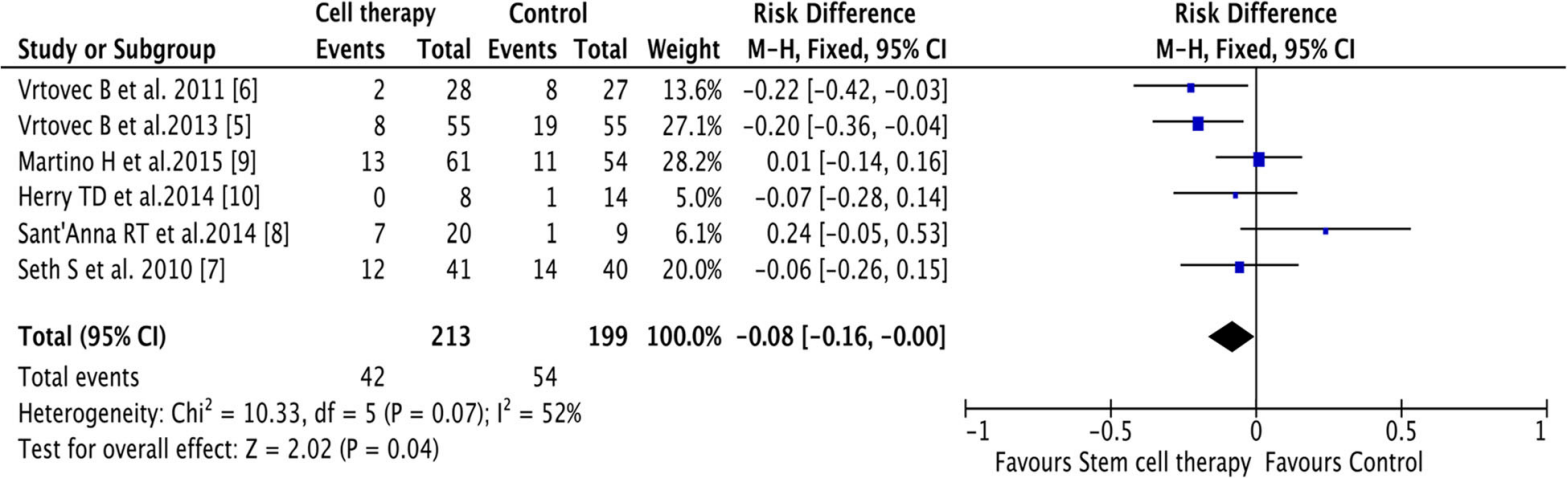
Mortality of stem cell therapy. Forest plot of the weighted mean differences revealed a reduced mortality rate after cell therapy compared with the control treatment. *CI* confidence interval

Meta-analytic pooling for mortality rate revealed that, compared with control treatment, bone marrow-derived stem cell therapy was associated with a significant decrease in mortality rate (19.7% (42/213) in the cell group vs. 27.1% (54/199) in the control group; 95% CI –0.16 to –0.00, *I* ^2^ = 52%, *p* = 0.04) (Fig. 2).

**Fig. 3:**
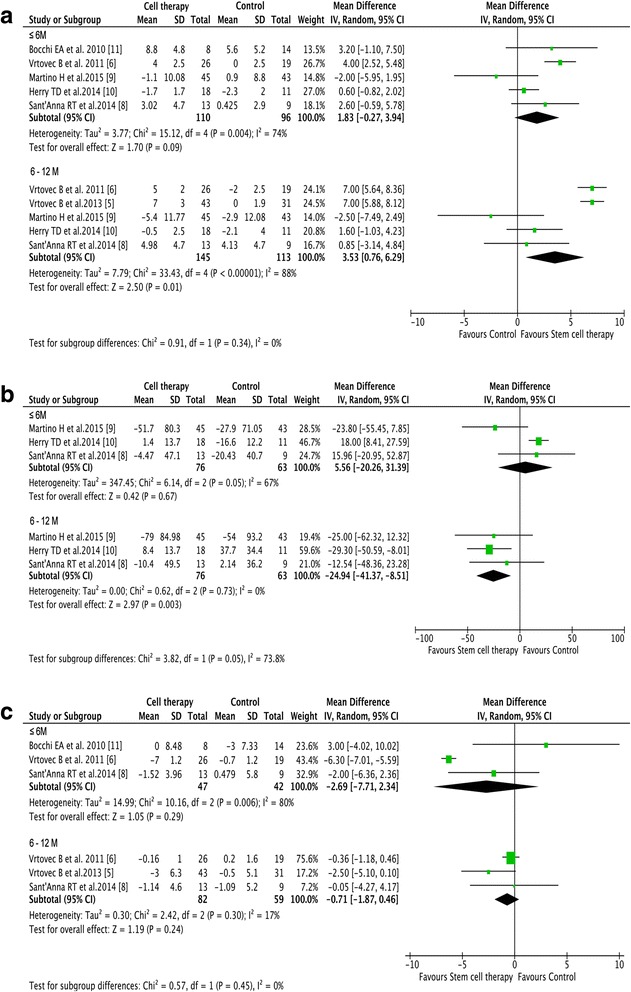
The effect of stem cell therapy on left ventricular structure and function. **a** Forest plot of the effect of stem cell therapy on left ventricular ejection fraction. **b**, **c** Forest plots of the effect of stem cell therapy on left ventricular end-systolic volume (**b**) and left ventricular end-diastolic chamber size (**c**). *CI* confidence interval, *IV* inverse variance, *SD* standard deviation

With respect to changes in LVEF, only a trend of improvement in LVEF was observed in the cell group at short-term follow-up (1.83% increase; 95% CI –0.27 to 3.94, *I* ^2^ = 74%, *p* = 0.09; Fig. 3a). However, compared with control treatment, bone marrow-derived stem cell therapy achieved significant improvement in LVEF at mid-term follow-up (3.53% increase; 95% CI 0.76 to 6.29, *I* ^2^ = 88%, *p* = 0.01) (Fig. 3b). Bone marrow-derived stem cell therapy reduced LVESV (–24.94 ml; 95% CI –41.37 to –8.51, *I* ^2^ = 0%, *p* = 0.003) after mid-term follow-up but did not influence left ventricular end-diastolic chamber size at any of the follow-up time points (–2.69 mm; 95% CI –7.71 to 2.34,﻿ *I* ^2^ = 80%, *p*﻿ = 0.29 at the short-term follow﻿﻿-up;–0.71 mm; 95% CI –1.87 to 0.46, *I* ^2^ = 17%, *p* = 0.24 at the mid-term follow-up) (Fig. 3c).

### Secondary outcomes

**Fig. 4:**
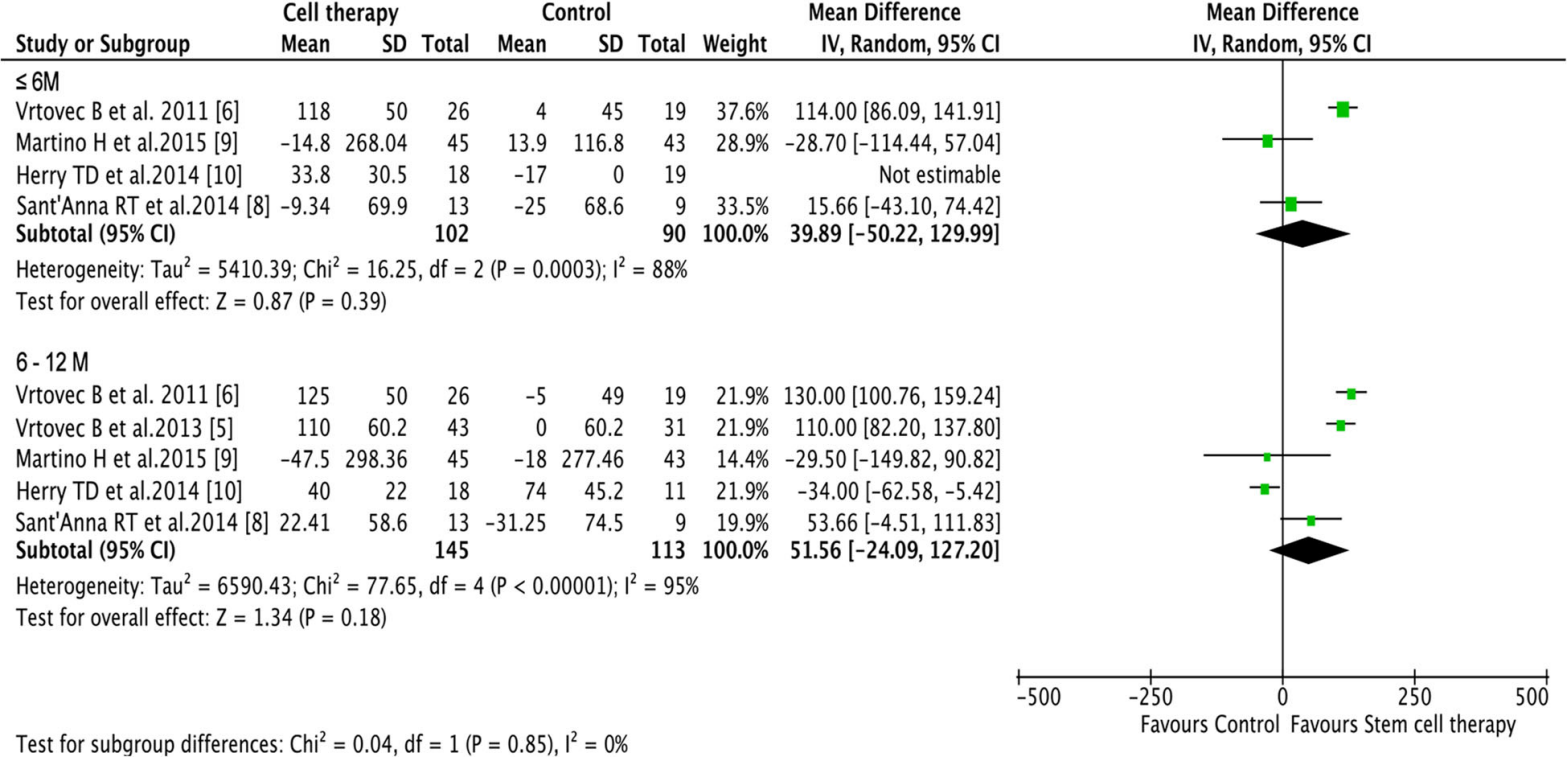
The effect of stem cell therapy on the 6-minute-walk test. Forest plots revealed no beneficial impact of stem cell therapy on the 6-minute-walk test compared with control. *CI* confidence interval, *IV* inverse variance, *SD* standard deviation

No significant improvement in 6MWT results was identified in the cell group compared with the control group at either follow-up duration (39.89 m increase; 95% CI –50.22 to 129.99, *I* ^2^ = 88%, *p* = 0.39 at the short-term follow-up; 51.56 m increase; 95% CI –24.09 to 127.20, *I* ^2^ = 95%, *p* = 0.18 at the mid-term follow-up) (Fig. 4).

### Sensitivity analysis and publication bias

A sensitivity analysis that excluded each individual study confirmed the significance of the mortality results obtained from the overall analysis. Additionally, no publication bias was found.

## Discussion

Based on currently available data, the main results of the meta-analysis are as follows: 1) Bone marrow-derived stem cell therapy may have reduced mortality rate and provided mild-to-moderate benefits in cardiac systolic function at mid-term follow-up for patients with NIDCM; 2) this therapy produced no observed improvement in exercise capacity.

NIDCM, an end-stage cardiomyopathy without coronary artery disease, often progresses into symptomatic HF due to a lack of effective pharmaceutics and interventional strategies. Patients with NIDCM exhibit a 31% mortality rate [17]. Thus, reducing this mortality rate is critical. At present, the major treatments for NIDCM are medication and heart transplantation. However, due to the limited curative effects and poor prognoses associated with these approaches, stem cell therapy has become a focus of clinical research [18].

In this systematic review, a promising result was obtained; mortality rate decreased more in the stem cell group than in the control group during 12 to 60 months of follow-up. With respect to left ventricular systolic function, stem cell therapy did not significantly improve LVEF until after mid-term (6–12 months) follow-up (a 3.53% increase in LVEF). Consistently, reduced end-systolic left ventricular chamber size was not observed until after 6 months of follow-up. We hypothesize that the “delayed” nature of LVEF enhancement might be due to cardiac remodelling mechanisms. Paracrine stem cell mechanisms, rather than mechanisms of the transplanted cells themselves, play important roles in LVEF improvement [19, 20]. Anti-inflammatory, immunomodulatory, and antioxidant activities play critical roles in reversing cardiac remodelling. Recent studies [21, 22] have demonstrated that stem cell transplantation can prevent remodelling and stimulate reverse left ventricular remodelling, a process that requires an average of 3 to 12 months. Research has suggested that, in addition to paracrine effects, other effects, such as the mircrine phenomenon, contribute to the therapeutic effects of stem cells. In this phenomenon, miR-499-overexpressing human cardiac stem cells are regarded as “donors”, and nearby cardiac stem cells serve as “recipients”. Observations of connexin 43 have suggested the potential translocation of miR-499 via gap junction channels. This new mode of cell-to-cell communication is known as mircrine communication, which also requires time to affect cells [23]. Interestingly, the results from cell therapy for ICM suggest a similar magnitude of LVEF improvement but more rapid LVEF recovery [16, 24, 25]. Differences in the onset time of cell therapy may be attributed to mechanisms that contribute to the progression of NIDCM and ICM. By definition, the most conspicuous difference between ICM and NIDCM is the existence of atherosclerotic lesions in the epicardial coronary arteries in patients with ICM and the absence of such lesions in patients with NIDCM. In theory, these lesions produce sufficient homing signals to induce stem cell engraftment in the damaged myocardium. Another abnormality in ICM is the presence of typically extensive dysfunctional areas of myocardial scarring; such regions are less pronounced or absent in patients with NIDCM [26, 27].

From our meta-analysis, we could not identify a beneficial effect of bone marrow-derived stem cell therapy on 6MWT results during follow-up. This finding might be attributable to the limited improvement in left ventricular systolic function (3.53%) observed in the cell group. This effect was statistically significant but clinically insufficient to produce a noticeable change in exercise capacity. Recent studies [28, 29] have reported that 6MWT distance is positively correlated with the magnitude of increase in cardiac output (*r* = 0.63), and inadequate output increase may therefore account for a lack of change in 6MWT results after stem cell therapy.

Certain research has revealed that the potential of autologous bone marrow-derived stem cell therapy for cardiac repair may be limited by patient-related factors, such as age and gender. Stem cell therapy in patients with acute myocardial infarction has been particularly effective at improving LVEF for ageing patients. This phenomenon likely occurs because ageing patients are likely to suffer from impaired endothelium and to exhibit inadequate physiological angiogenesis responses to ischaemia; thus, such patients will tend to greatly benefit from supplementation with stem cells [30]. Gender may be a significant determinant of stem cell function via the direct actions of sex steroids on these cells. A prior study found greater post-ischaemic myocardial functional recovery after an intracoronary infusion of female stem cells than after an intracoronary infusion of male stem cells [31].

### Limitations

Significant heterogeneity was noted among the trials with respect to left ventricular function and quality of life. This heterogeneity was due to multiple factors, including the number of patients enrolled, whether trials were single-centre or multicentre studies, the number of cells injected, routes of injection, and the use of granulocyte colony-stimulating factor (G-CSF) therapy in three trials [5, 6, 9]. The cell types used in the included trials are limited to BM-CD34 cells and BMMNCs. Nonetheless, because three original papers involving 178 patients used BM-CD34 cells as the cell source and four trials involving 304 patients used BMMNCs, additional analyses would have been unreliable. Second, in this study, cardiac function referred only to systolic function. When more data are available, it would be interesting to explore the efficacy of stem cells for improving diastolic function.

## Conclusions

Bone marrow-derived stem cell therapy may have produced a net beneficial effect with respect to reducing mortality in patients with NIDCM. When such therapy was utilised, a mild-to-moderate LVEF increase was only observed during mid-term follow-up, with no improvement in exercise capacity. Future research should focus on the most effective cell type and dosage for stem cell therapy.

## Abbreviations

6MWT: 6-minute-walk test
BMMNC: Bone marrow mononuclear cell
CI: Confidence interval
HF: Heart failure
ICM: Ischaemic cardiomyopathy
LVEDD: Left ventricular end-diastolic dimension
LVEF: Left ventricular ejection fraction
LVESV: Left ventricular end-systolic volume
NIDCM: Non-ischemic dilated cardiomyopathy
RCT: Randomised controlled trial
WMD: Weighted mean difference
